# Utilization of Isometric Strength Training and Interval Training With a Patient With Cancer in the Acute Care Setting

**DOI:** 10.7759/cureus.15570

**Published:** 2021-06-10

**Authors:** Andrew Chongaway

**Affiliations:** 1 Physical Therapy, Beaumont Health, Royal Oak, USA

**Keywords:** mesothelioma, physical therapy, high intensity interval training, acute care, exercise oncology

## Abstract

The onset of cancer and subsequent treatments often result in deficits in physical function and quality of life (QoL). Available research has demonstrated that an individualized exercise program has the ability to reduce fatigue, optimize physical function, and improve QoL. However, the exercise program is often lacking appropriate intensity and volume resulting in negligent improvement or even further reduction in function. Thus, the purpose of this case report is to demonstrate the application of isometric strength training (IST) and high-intensity interval training (HIIT) in the acute care setting with an individual with a history of malignant mesothelioma. The patient demonstrated improvement in functional mobility evidenced by improvement in Activity Measure for Post-Acute Care (AM-PAC) score through the hospitalization along with increased ambulation distance. No adverse events occurred during any physical therapy (PT) visits while utilizing HIIT or IST.

## Introduction

Malignant mesothelioma is a cancer that can develop from exposure to and inhalation of asbestos, which is a known carcinogen that was frequently used in building materials [1]. Since the early 2000s, the incidence of malignant mesothelioma has decreased, however, within the United States there are still roughly 3,000 new cases each year [2]. Of those cases, the majority are men in their 50-70s [1,2]. Men are 4.5 times more likely to develop malignant mesothelioma due to the high prevalence of exposure occurring in industrial and textile factory settings [1]. The mesothelium is a special lining comprised of mesothelial cells that cover numerous major organs. The mesothelial cells secrete a lubricating fluid that allows the free movement of the organs against neighboring organs (i.e., the pleura covering the lungs allows free movement of the lungs around the heart when expanding or contracting) [2]. Other examples of mesothelial linings include the pericardium which surrounds the heart, the peritoneum which lines the abdomen, and tunica vaginalis which lines the testicles [1,2]. The majority of all malignant mesothelioma diagnoses are malignant pleural mesotheliomas [2]. There are three histological types of malignant mesothelioma: epithelial, fibrous, and biphasic type, with the epithelial type being the most commonly diagnosed (~50%) and carries a more favorable prognosis [2].

The progression of malignant pleural mesothelioma ranges between 20 and 30 years and usually affects the right side more compared to the left [1]. Individuals will usually present with chest pain in the distal and posterolateral aspect of the thorax, dyspnea, hypertrophy of the opposite hemithorax, pleural effusion, fatigue, insomnia, and cachexia [1,3]. These individuals will often have a high symptom burden at the time of diagnosis with only about 25% of patients demonstrating metastatic disease [1].

Due to the location and nature of malignant mesothelioma, surgery and the use of radiation therapy remains controversial [1]. However, for individuals who are diagnosed with medically operable stages I to IIIA disease, have limited co-morbidities, and display appropriate pulmonary and cardiac function based on pulmonary function tests and a cardiac stress test, a multimodal treatment approach of surgery, radiation therapy, and chemotherapy may be appropriate [1,3]. With resectable malignant pleural mesothelioma, there are two standard surgical options that may be used: extrapleural pneumonectomy and pleurectomy with decortication [1]. Chemotherapy may be utilized in the neoadjuvant and adjuvant settings to reduce the tumor bulk and improve the chances for successful macroscopic tumor resection along with reducing the chance for recurrence [1]. Common first-line systemic treatment options are pemetrexed and cisplatin/carboplatin for six cycles followed by maintenance bevacizumab [1]. Due to the sensitive areas in the thorax (lung and heart), radiation therapy is utilized sparingly [1,3].

Due to the global impact of the disease and resulting treatment, individuals may experience decreased function and activity tolerance following surgery. These deficits may persist long-term, especially with the addition of systemic therapy or advanced disease progression. The reduction in physical function can lead to muscle atrophy, weakness, impaired functional capacity, frailty, increased risk of falls, and an overall reduced quality of life (QoL) [3]. This muscle atrophy can be defined as sarcopenia or cachexia, both of which are associated with a cancer diagnosis. Sarcopenia is a reduction in muscle quality and neurodegeneration, leading to reductions in strength, aerobic capacity, and metabolism. Cachexia is a multifactorial syndrome defined by loss of skeletal muscle mass with or without fat wasting that cannot be reversed by nutrition support in the context of chronic systemic inflammation and metabolic alterations [4].

Exercise has been demonstrated to promote improvements in physical function by increasing skeletal muscle strength and aerobic capacity in the pre-operative and post-acute/advanced disease setting. With regular exercise, survivors of mesothelioma have shown increased function, independence, and reduced effects of the tumor burden and treatments [5,6]. High-intensity interval training (HIIT) has been demonstrated to be safe with older and at-risk populations and can be adapted appropriately with most individuals [6]. Furthermore, HIIT has the ability to be carried out with bodyweight or resistance and can be completed in varying positions. This is beneficial to easily promote activity throughout the day during hospitalization [6]. Unfortunately, in the acute care setting, cancer survivors may lack the functional reserve, strength, balance, or energy to safely produce the appropriate stimulus with standing, dynamic, or HIIT exercises to promote functional gains. In these situations, isometric strength training (IST) has been demonstrated to be an appropriate alternative to promote strength gains with lower energy demand [7]. Unfortunately, exercise prescription especially in the acute care setting is often sub-threshold in terms of intensity and volume and lacks individualization [8]. IST and HIIT are two viable options to utilize in the acute care setting and promote adequate stimulus and intensity [6,7]. The purpose of this case report is to demonstrate the application of IST and HIIT in the acute care setting with an individual suffering from a history of malignant mesothelioma.

## Case presentation

Patient history and systems review

The patient consented to participate in this case report. The patient was an 80-year-old male who was admitted to an Acute Care Community Hospital (ACCH) after presenting to the emergency department with concerns of a fever, cough, dyspnea, lower extremity weakness, and fatigue that had started roughly two weeks prior to admission. He had recently restarted maintenance chemotherapy, pemetrexed (Alimta), one week prior to admission following a chemotherapy holiday directed by his medical oncologist due to loss of appetite and weight loss.

**Figure 1:**
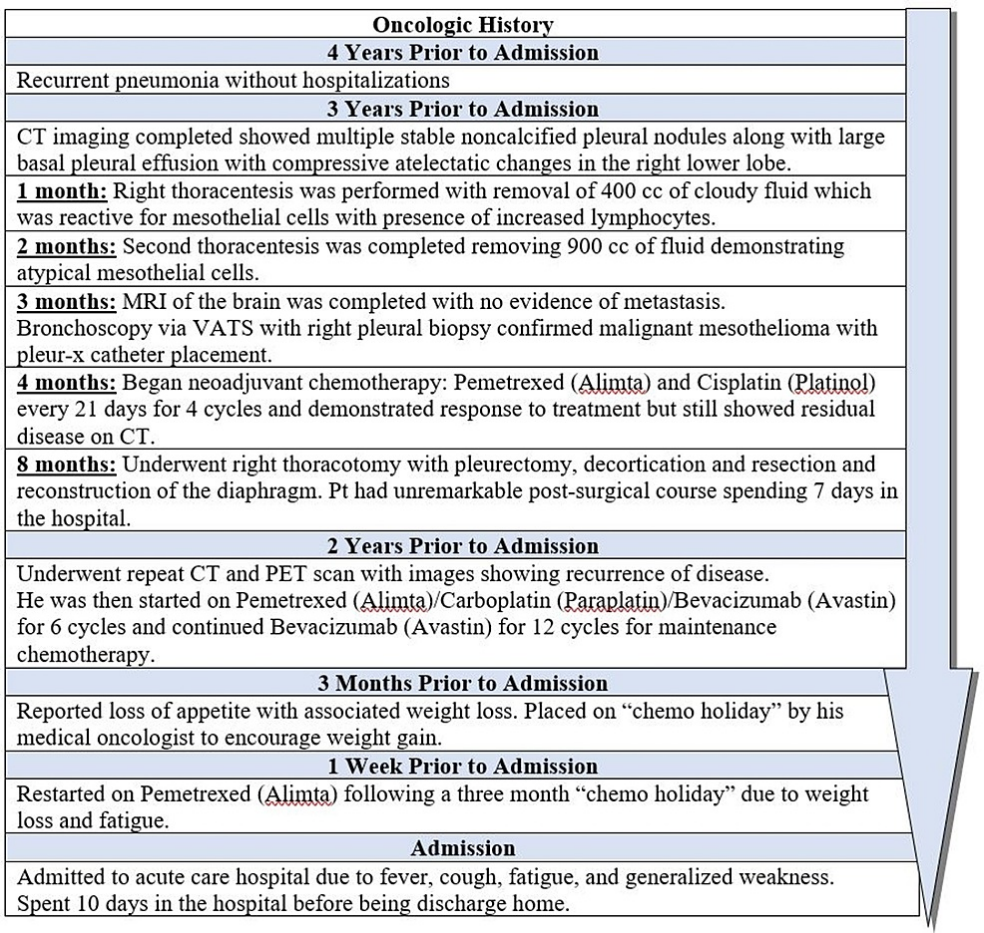
Oncologic history timeline CT: computed tomography; MRI: magnetic resonance imaging; VATS: video-assisted thoracoscopic surgery; PET: positron emission tomography.

**Figure 2:**
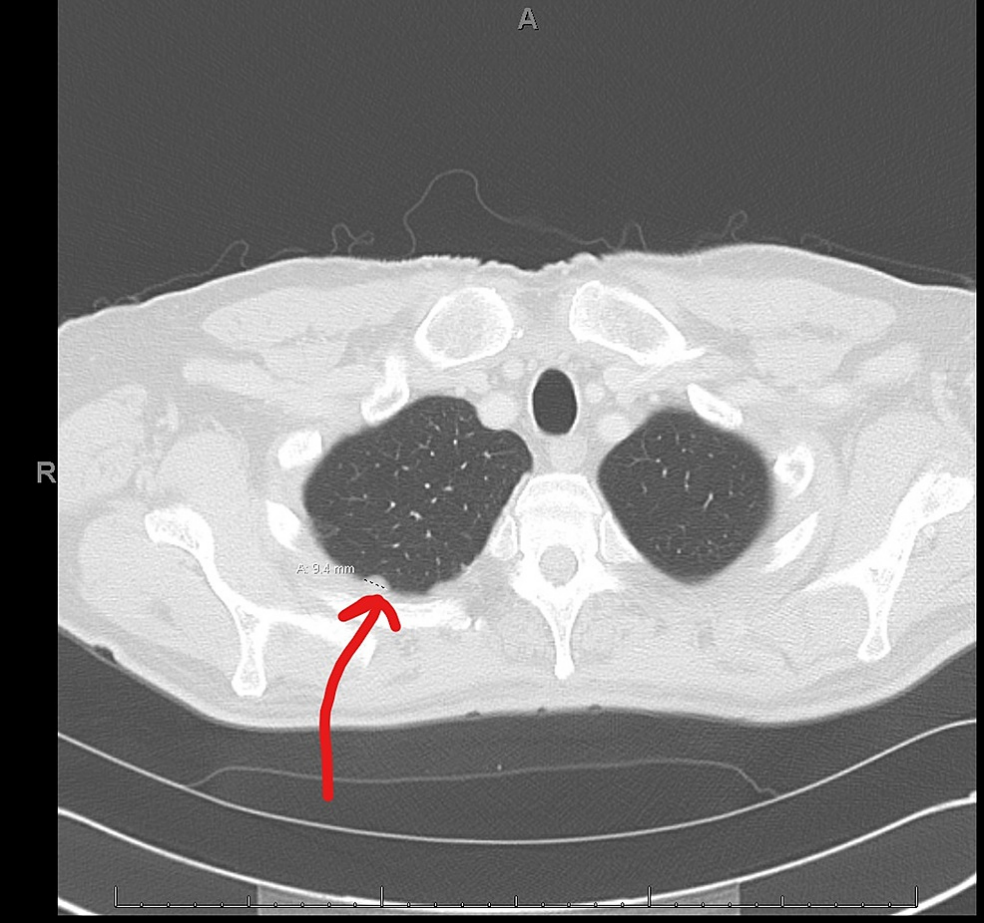
Preoperative chest CT following neoadjuvant chemotherapy demonstrating 9.4 mm nodule within the right lung CT: computed tomography.

**Figure 3:**
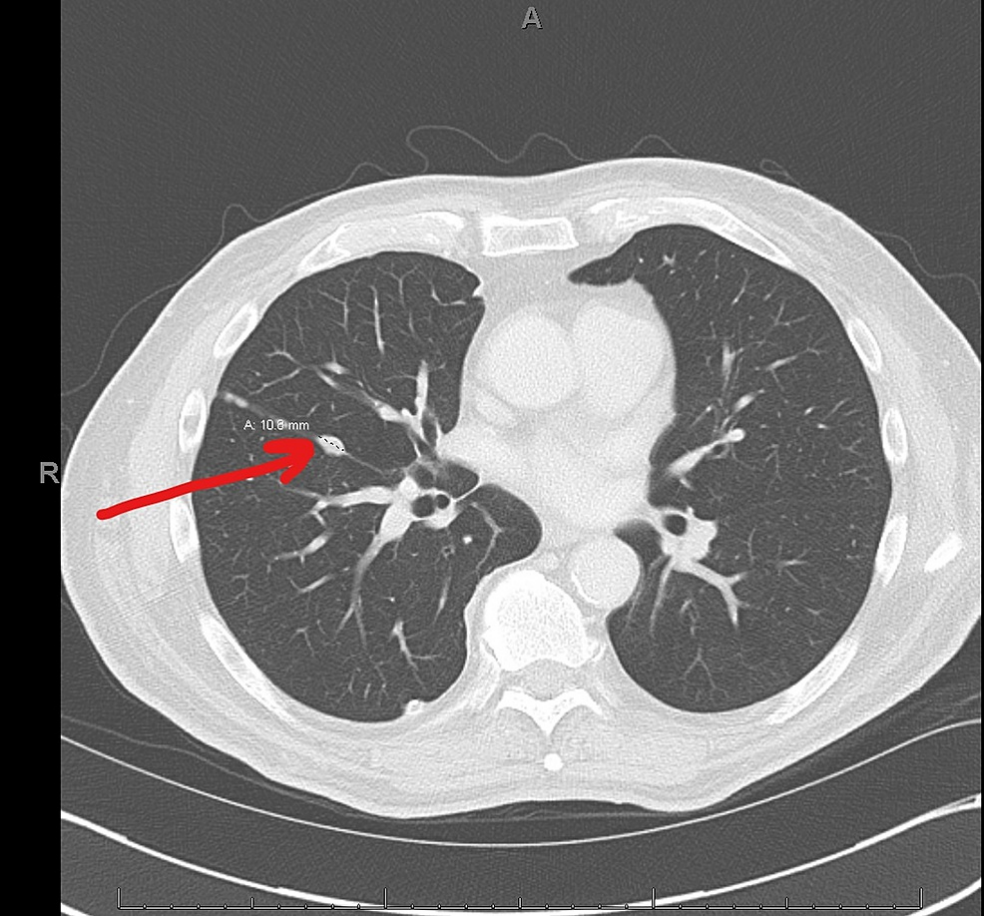
Preoperative chest CT following neoadjuvant chemotherapy demonstrating 10.8 mm nodule in the right lung CT: computed tomography.

**Figure 4:**
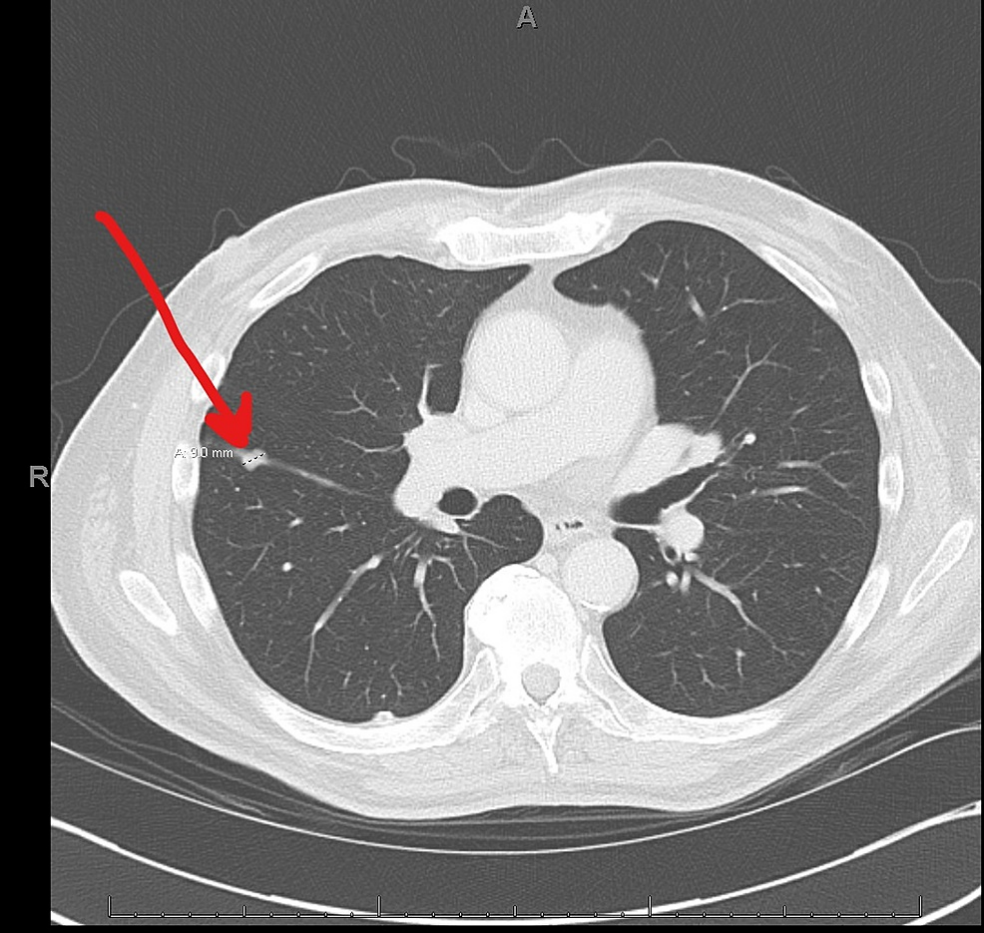
Preoperative chest CT following neoadjuvant chemotherapy demonstrating 9 mm nodule in the right lung CT: computed tomography.

**Figure 5:**
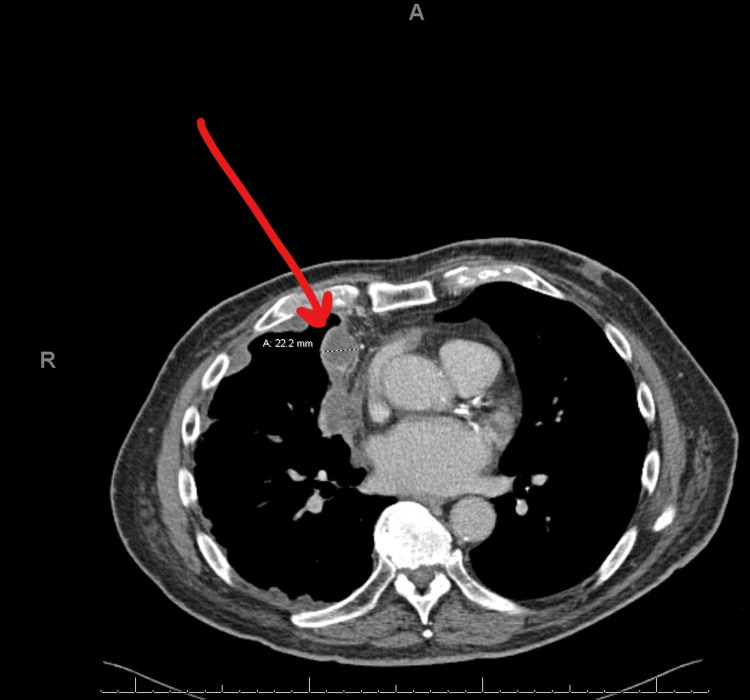
Chest CT examining the progression of the disease with the pleural-based mass measuring 2.3 cm × 4.4 cm abutting the right atrium CT: computed tomography.

**Figure 6:**
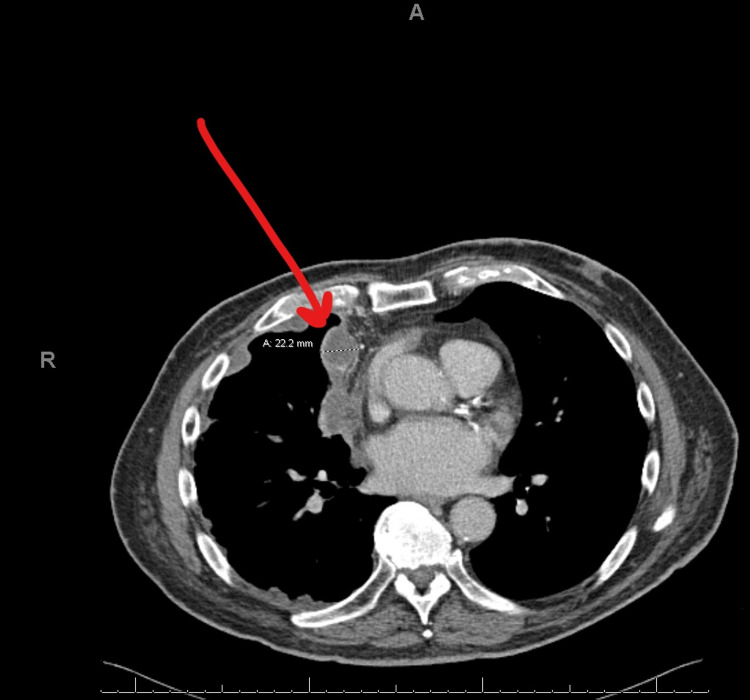
Chest CT examining the progression of the disease with the pleural-based mass medially to the right middle lobe measuring 2.2 cm CT: computed tomography.

The patient was previously diagnosed with stage II epithelioid subtype malignant mesothelioma of the right lung three years prior to this admission. At the time of diagnosis, the patient underwent neoadjuvant chemotherapy, pleurectomy and decortication of the right pleura and diaphragm, and adjuvant/maintenance chemotherapy. The timeline of his oncologic history is shown in Figure 1. Table 1 provides a more concise description of his oncologic treatment history including surgical procedures and chemotherapy regimens. Pre-operative and post-operative imaging are shown in Figures 2-6. The patient’s past medical history was also significant for hypertension, coronary artery disease, stage III chronic kidney disease, and anemia. Aside from the pleurectomy and decortication, his past surgical history was significant for multiple thoracenteses, placement of a Pleur-x catheter, and a biceps tendon rupture repair of the right upper extremity.

**Table 1:** Oncologic treatment history

Oncologic treatment history					
Region	Pathology	Staging	Surgery	Systemic therapy	Radiation therapy
Right lung	Malignant mesothelioma: epithelioid subtype	Stage II	Right thoracotomy with pleurectomy, decortication, resection, and reconstruction of the diaphragm.	Neoadjuvant chemotherapy: pemetrexed (Alimta) and cisplatin (Platinol) for four cycles. Adjuvant chemotherapy: pemetrexed (Alimta), carboplatin (Paraplatin), and bevacizumab (Avastin) for six cycles. Maintenance chemotherapy pemetrexed (Alimta) for 12 cycles. Following the progression of the disease was started on gemcitabine (Gemzar) for six cycles.	N/A

As per the chart review, the patient had a painful rash located on his chest which had been ongoing for two weeks, developing after he had been started on gabapentin (Neurontin). Prior to this admission, he had worked as a lawyer and lived with his wife in a two-story home with the bedroom and bathroom located on the second floor. He did have a prior smoking history of half a pack a day for three years, though he reported quitting over 50 years ago. He also denied any direct exposure to asbestos but did state his father worked in the textile industry when he was younger and may have had incidental exposure. His family history was significant for his father and sister having lung cancer and his mother having heart disease. At the time of admission, the patient was taking amlodipine (Norvasc) and ezetimibe (Zetia). The patient reported enjoying walking and golfing but had been unable to in several weeks due to weakness and fatigue.

During the physical therapy (PT) consult, the patient expressed concerns of gait instability as he reported a fall one month prior when walking his dogs. He denied hitting his head during the fall or breaking any bones. The patient reported limited activity in the two weeks prior to the admission due to increased fatigue, generalized weakness, and increased fear of falling. Due to the previous fall and onset of fatigue and weakness, the patient expressed concern about a loss of independence with activities of daily living (ADLs) and a reduced QoL as the patient no longer walked his dog and only completed limited activities around his house. He expressed that with working with PT he would like to work towards being able to walk his dog again and feel more confident with his balance. A complete systems review is given in Table 2.

**Table 2:** Systems review ROM: range of motion; WFL: within functional limits.

Systems review		
Cardiovascular/pulmonary	Vitals took in sitting: Blood pressure: 124/60 mmHg, heart rate: 79 beats per minute, peripheral capillary oxygen saturation (SpO_2_): 94% on room air. No signs of tachypnea or labored breathing	
Musculoskeletal	No peripheral edema, no joint pain/swelling, no evidence of recent injuries. Tended to adopt kyphotic posture with unsupported sitting or standing. ROM; WFL bilaterally strength; minimal weakness noticed bilaterally in lower extremities.	
Neuromuscular	An intact light touch to bilateral hands with diminished light tough in bilateral feet (patient endorsed numbness/tingling in bilateral toes).	
Integumentary	Integrity: Unimpaired skin color/discoloration: rash; red located on anterior chest. Well-healed surgical scars across apparent on abdomen with minimal fascial restriction.	
Lab values	White blood cells	3.2 × 10^9^L (reference value: 5.0-10.0 × 10^9^/L)
	Hemoglobin	10.7 g/dL (reference value: male: 14-17.4 g/dL)
	Hematocrit	33.5% (reference value: male: 42-52%)
	Platelets	146 k/µL (reference value: 140-400 k/µL)
Communication	Unimpaired	
Affect, cognition, language, learning style	The patient was alert and oriented to person, place, and time. Affect appropriate English language ability to learn through visual demonstration, auditory, and written information.	

This patient was chosen for a case study due to his significant history of malignant mesothelioma accompanied by surgical intervention and extensive history of systemic therapy with functional mobility deficits to demonstrate the utilization of IST and HIIT in the acute care PT setting.

Examination - tests and measures

At initial evaluation (IE), manual muscle testing (MMT), gross range of motion (ROM), and sensation to light touch were assessed along with bed mobility, transfers, and gait. The timed up and go (TUG) and Activity Measure for Post-Acute Care basic (AM-PAC) mobility 6 clicks were used as outcome measures [9-12].

The MMT revealed good (4/5) strength of bilateral lower extremities. Gross ROM was assessed with the patient having within functional limits (WFL) active ROM (AROM) of bilateral upper extremities and lower extremities, though left shoulder flexion was slightly limited compared to the right upper extremity. He also demonstrated intact sensation to bilateral upper extremities but had diminished light touch sensation of bilateral lower extremities. He reported 5/10 burning pain at his anterior chest based on the Numeric Pain Rating Scale (NPRS) due to the rash [13]. He required supervision with sit-to-stand transfers from the bed and chair with the use of upper extremities for support and minimal physical assistance with bed-to-chair transfers due to unsteadiness with turns. He was able to ambulate for 49 m (160 ft) without an assistive device (AD), though had two lateral losses of balance (LOB), requiring moderate assistance to correct both times. The patient completed the TUG without an AD for two trials, requiring 25 and 23 seconds to complete with an average time of 24 seconds, indicating an increased risk for falls [11]. During both trials, he demonstrated hesitancy with turning with a slight lateral loss of balance during each turn requiring external support for safety. Lastly, the patient scored 19/24 on the AM-PAC Basic Mobility 6 clicks (6=lowest functional score, 24=highest functional score), indicating that the patient was safe to return home with no services, though due to his concerns with fatigue, weakness, and loss of QOL, outpatient PT was also recommended [12]. The duration of the PT's initial evaluation was 45 minutes. Detailed information regarding patient performance during IE and subsequent visits are given in Table 3.

**Table 3:** Physical function AD: assistive device; RW: rolling walker; AM-PAC: activity measure post-acute care basic mobility.

Physical function				
Functional mobility	Visit 1 (completed at 1410)	Visit 2 (completed at 0940)	Visit 3 (completed at 1025)	Visit 4 (completed at 1125)
Bed mobility	Modified independent	Modified independent	Modified independent	Independent
Transfers: sit to stand	Supervision or setup	Supervision or setup	Independent	Independent
Transfers: bed to chair	Minimal assistance	Minimal assistance	Supervision or setup	Supervision or setup
Gait	Moderate assistance	Moderate assistance	Supervision or setup	Supervision or setup
Stairs	Not assessed (the patient reported significant difficulty with stairs in recent weeks)	Not assessed	Not assessed	Supervision or setup
Distance ambulated	49 m (160 ft) without AD; 3 × 6 m^2^ (20 ft) without AD with two losses of balance	3 × 7.5 m^2^ (25 ft) with RW	7.5 m (25 ft) without AD	76 m (250 ft) with RW
AM-PAC 6 clicks	19/24	19/24	20/24	23/24
Performance status				
Timed up and go	25/23 seconds			
Berg Balance Scale				46/56

Clinical impression

Though the AM-PAC 6 clicks predicted that the patient was safe to return home with no services, it was determined the patient would benefit from skilled PT services while hospitalized as the patient was below a baseline of his prior level of function (PLOF). Additionally, while admitted, it was anticipated that the patient may continue to demonstrate a decline in functional mobility due to limited ability to participate in out-of-bed activity throughout the day [13]. Deficits in nutrition, recent weight loss, lower extremity weakness, limited functional reserve, and impaired balance were evidenced by the patient ambulating 49 m (160 ft), scoring well below normative values on the TUG, and demonstrating two LOBs during ambulation [14]. These deficits placed the patient at a higher risk for falls and facilitated the need for assistance with ADLs and impaired QoL.

The proposed plan of care (POC) to address the patient’s deficits was to include therapeutic exercise to promote lower extremity strength and endurance, neuromuscular re-education to promote static and dynamic standing balance, as well as anticipatory and reactive balance, and gait training with and without an AD to facilitate safe mobility within the home and community.

Of note, the patient subjectively reported potential measles exposure three days after being admitted limiting the ability to ambulate outside of the room. The patient was then placed in airborne isolation precautions for four days. Due to the patient consistently reporting fatigue and weakness, an individualized exercise prescription was developed to assist the patient in making progress towards his PLOF as he was motivated to participate with PT and had been previously independent with ADLs.

Intervention and plan of care

The patient was scheduled for skilled PT sessions two to four times per week. This frequency was determined based on facility protocol and that the patient was of higher functional status and would be able to complete a home exercise program (HEP) independently. The patient’s care was coordinated with OT and nursing. The patient education at the IE and subsequent visits consisted of the benefits of exercise in regard to fatigue, strength, and functional mobility. The patient was educated and instructed on the use and benefit of an AD to promote safety with ambulation. During each visit, functional mobility and AM-PAC 6 clicks were assessed. The patient was educated on the Visual Analog Scale for Fatigue and Rate of Perceived Exertion (RPE) [15,16]. The scales were used to appropriate dose type, time, intensity, and volume of the interventions for each visit [15,16].

Directly following the IE, the patient was instructed in exercises focused on the recruitment of key muscles used during functional activities with exercises being performed both in supine and standing. After receiving the HEP, time was spent reviewing the handout for the understanding of the exercise description as well as when to perform the HEP, how many sets and repetitions to complete, rest time between sets, and when to adjust or stop exercising due to safety concerns (e.g., lightheadedness, vomiting, new onset of pain). Lastly, time was spent educating the patient on the difference between muscle soreness and pain to promote adherence and understanding of physiological processes. As he did not experience pain during the initial evaluation with exercises, it was explained that he may experience soreness the following day(s) after exercising which may be relieved with further activity, exercise, or stretching whereas pain may linger and become worse with further activity, exercise, stretching.

For visits 2 and 3, ambulation and exercises were completed in the patient’s room due to the patient being placed in airborne isolation precautions. Exercises were first demonstrated, then the parameters of each exercise(s) were explained. For visit 2, the patient reported 6/10 fatigue at the start of the session. Multiple small walks were completed in the room with the use of a rolling walker (RW) with the patient demonstrated improved safety and no LOBs during room ambulation. Exercises within the visit focused on seated and standing reactive and anticipatory balance, and proximal lower extremity strength via IST and HIIT. No adverse events were noted. He rated the isometrics and balance exercises between 10 and 12/20 and the HIIT style exercises between 15 and 16/20 with an overall RPE of 13/20 (somewhat hard), and the session lasted 30 minutes.

At the start of visit 3, the patient reported 3/10 fatigue. Since there was subjectively reduced fatigue, a HIIT style visit was utilized to promote muscular strength and endurance with elements of anticipatory and reactive balance incorporated into the session [17]. No adverse events were observed during the session. At the end of the visit, the patient reported an RPE of 15/20 (hard), and the session lasted 30 minutes.

For visit 4, the patient was cleared of the diagnosis of measles and was no longer under airborne precautions. The visit consisted of gait training with an RW within the halls. The session focused on efficiency and safety with navigation of turns and obstacles, and fall risk/balance assessment via the Berg Balance Scale (BBS) [18]. The patient scored 46/56 (56=highest balance score), indicating a low risk of falls. The visit lasted 25 minutes and the patient reported an RPE of 10/20. A detailed description of interventions per visit is given in Table 4.

**Table 4:** Exercise prescription

Exercise Program per Visit	
Rx visit 1	
Sidelying hip abduction	2 sets of 10 each leg
Supine bridges	2 sets of 10
Standing hip abduction with resistance	2 sets of 10 each leg
Standing hip extension with resistance	2 sets of 10 each leg
Mini squats	2 sets of 10
Standing single-leg heel raises	1 set of 10 each leg
Rx visit 2	
Seated balance at the edge of the bed against moderate-maximal perturbations	1 set of 2 minutes
Seated hip flexion isometrics (Alt. each leg)	1 set of 10 with a 10-second hold on each leg
Seated hip abduction Isometrics	1 set of 10 with a 10-second hold on each leg
Seated knee extension isometrics (alt. each leg)	1 set of 10 with a 10-second hold on each leg
Seated knee flexion isometrics (alt. each leg)	1 set of 10 with a 10-second hold on each leg
Supine bridges	4 rounds: 30 seconds of work with 2 minutes rest
Sit to stands	4 rounds: 30 seconds of work with 2 minutes rest
Standing balance reaching across midline	1 set of 20
A close stance against moderate perturbations	2 sets of 2 minutes
Rx visit 3	
Standing marches in place and tandem walk	3 rounds: 30 marches into a 20-foot tandem walk with 2-minute rest between rounds
Squats and close stance eyes open	2 rounds: 10 squats into 1-minute close stance eyes open with 1-minute rest between rounds
Forward marching and mini lunges	2 rounds: 20 forward marches into 10 min lunges with 2-minute rest between rounds
Single leg balance	5 sets of 5 seconds on each leg
Stagger stance against moderate perturbations	2 sets of 2 minutes
Rx visit 4	
Berg balance scale and gait training	
Four rounds of squats - standing resistance band rows - lunges - wall push-ups	Each exercise completed for 30 seconds with a 30-second rest break between each exercise. After each round of wall push-ups, a 2-4 minute rest break was given depending on the patient’s report of readiness for the following round.

Outcomes

The patient was seen for four visits over seven days. He was discharged from the hospital before a formal reassessment of the goals could be completed. However, he did show improvement with functional mobility compared to the initial evaluation (Table 3). At the time of discharge, he demonstrated independence or supervision with functional mobility tasks resulting in an increase in AM-PAC “6 clicks” from 18/24 on visit 1 to 23/24 on visit 4. During visit 4, he also ambulated for an additional 27.5 m (90 ft) with no reported LOBs with the use of an RW.

Overall, the patient demonstrated improved functional mobility and safety with gait. The patient was educated on the differences between the RW and a four-wheeled walker with the patient preferring the RW due to the ease of transport and only requiring the use of an AD for a short period of time after discharge. He also agreed to continue to follow the established HEP. He also did express interest in outpatient PT. However, at the time of publication, he had not participated in any skilled outpatient PT. As previously mentioned, no adverse events were noted or reported following each visit.

## Discussion

The purpose of this case report was to demonstrate the application of IST and HIIT in an acute care setting for a patient with a history of malignant mesothelioma who had an extensive adjuvant treatment history. No adverse events occurred during the visits and were adapted and modified based on the patient's report of fatigue each visit.

As previously stated, muscle atrophy as well as other adverse effects such as fatigue, sedentarism, and poor nutrition status is associated with a reduced QOL in persons living with and beyond cancer (PLWB) [3,4,8,19]. With significantly limited activity time during hospitalization, patients experience additional muscle atrophy, resulting in further decline in function and leading to increased dependence and a higher chance for readmission upon discharge [8]. This decline in function and functional capacity while hospitalized was termed hospital-acquired deconditioning (HAD) [8]. While PT has the ability to promote functional gains, the interventions prescribed are often sub-threshold and generalized, resulting in little to no benefit for the patient [8,20]. Appropriate frequency, intensity, and volume are required for the exercises to be beneficial and promote physiologic change, including muscle protein synthesis, mitochondrial biogenesis, and promotion of antioxidant effects. The lack of activity time for patients in the hospital, along with the generalized and often deficient exercise prescription, often promotes muscle atrophy, functional decline, and HAD [4,8,20].

Falvey et al. discussed the need to change the thought process and direction of PT care in the acute care setting to promote optimal outcomes and reduce the risk of subsequent impairments and readmission [8]. Two statements made in the article that hold substantial weight are defining the term “General Conditioning Activities” (GCAs) and discussing the need to shift the focus from GCAs and sub-threshold exercise to a more precise, individualized exercise prescription utilizing moderate to high intensity aerobic, resistance, and balance exercises. The term GCA is defined as hallway ambulation, non-specific active range of motion exercises, and group exercises that are sub-threshold with no substantial attempt to individualize appropriate resistance, volume, intensity, and or progressive overload [6,8,20]. Second, restructuring the rehabilitation hierarchy for older adults is required to reduce the risk of developing HAD. The author recommends a focus on high-intensity resistance training and moderate to high-intensity motor control-based gait, balance, and ADL training compared to GCAs and simple gait, balance, and ADL training in the acute care setting [8]. By changing the focus of interventions and appropriately prescribing intervention intensity, patients’ physical function may be maintained or improved, resulting in a shorter length of stay, improved QOL, and a reduced risk for readmission or need for assistance following discharge [8].

Through research, it is known that exercise is beneficial for cancer survivors in any setting. This understanding was expanded in 2019 when the American College of Sports Medicine (ACSM) released new exercise guidelines for cancer survivors. The guidelines recommended a minimum of 30 minutes of moderate-intensity aerobic exercise three times a week and resistance training two to three times a week at 60% of one-repetition maximum for 8 to 15 repetitions [20]. These guidelines of combined modality demonstrated improvements in fatigue levels, overall physical function, and QoL [20]. Time and equipment may be limited in the acute care setting to meet these guidelines. Fortunately, with HIIT and IST no equipment is required though if resistance bands, light dumbbells, or aerobic machines are available can be utilized within the exercise program. For these reasons, IST and HIIT are still viable options to provide appropriate stimulus to patients of varying statuses to meet the recommended exercise guidelines.

Limitations and future research

There were several limitations to this case study. First, during visit 1, performance on stairs was not assessed but was assumed based on the patient's report of difficulty at home with stairs, and gait assessment was conducted without an AD. These two variables potentially resulted in a lower AM-PAC Basic Mobility score for visit 1 which resulted in a large change of AM-PAC Basic Mobility score between visits 1 and 4. Second, outcome measures were not reassessed during PT visits. During visit 1, the timed up and go was completed but was not reassessed during subsequent visits. The second outcome measure, the Berg Balance Scale, was completed during visit 4 and did demonstrate there was a low risk of falls though a re-test was not possible due to hospital discharge. A possible solution to promote reassessment of outcome measures would be to standardize the sequence of outcome measures in the acute care PT setting so that balance, functional capacity, and strength are measured at initial evaluation and would allow for consistent reassessment at given points during care.

## Conclusions

Traditional implementations of generalized exercises and sub-threshold activities limit the overall benefit for persons with cancer in the acute care or outpatient setting. The FITT principles (frequency, intensity, type, time) along with volume, pattern, and progression need to be considered when developing and implementing interventions for a patient. As stated previously, time and equipment are often voiced as major barriers in the acute care setting for appropriate stimulus and intensity. However, IST and HIIT so far in the literature have demonstrated positive effects in promoting the appropriate stimulus to maintain/increase muscle power, strength, and endurance. HITT also has the benefit of providing stimulus to promote cardiorespiratory function and can be completed in a multitude of positions (supine, sitting, and standing) depending on the patient’s functional status. By being precise with exercise prescription and challenging the patients, the chance for HAD and significant impairments may be reduced, resulting in an improved QOL and lower chance for readmission. As more research continues to be done with cancer survivors, a focus on exercise prescription (FITT) along with the use of IST and HIIT in the acute care setting is needed to provide clinicians with a framework and reference to promote optimal outcomes and instill change in their systems for their patient’s benefit.
